# An Exploration of Formal and Informal Mindfulness Practice and Associations with Wellbeing

**DOI:** 10.1007/s12671-018-0951-y

**Published:** 2018-05-21

**Authors:** Kelly Birtwell, Kate Williams, Harm van Marwijk, Christopher J. Armitage, David Sheffield

**Affiliations:** 1 University of Manchester, Manchester, England; 2 NIHR School for Primary Care Research, Manchester, England; 3 Brighton and Sussex Medical School, University of Brighton, Brighton, England; 4 NIHR Greater Manchester Patient Safety Translational Research Centre, Manchester, England; 5 University of Derby, Derby, England

**Keywords:** Mindfulness, Wellbeing, Psychological flexibility, Stress, Survey, Content analysis

## Abstract

Mindfulness has transdiagnostic applicability, but little is known about how people first begin to practice mindfulness and what sustains practice in the long term. The aim of the present research was to explore the experiences of a large sample of people practicing mindfulness, including difficulties with practice and associations between formal and informal mindfulness practice and wellbeing. In this cross-sectional study, 218 participants who were practicing mindfulness or had practiced in the past completed an online survey about how they first began to practice mindfulness, difficulties and supportive factors for continuing to practice, current wellbeing, and psychological flexibility. Participants had practiced mindfulness from under a year up to 43 years. There was no significant difference in the frequency of formal mindfulness practice between those who had attended a face-to-face taught course and those who had not. Common difficulties included finding time to practice formally and falling asleep during formal practice. Content analysis revealed “practical resources,” “time/routine,” “support from others,” and “attitudes and beliefs,” which were supportive factors for maintaining mindfulness practice. Informal mindfulness practice was related to positive wellbeing and psychological flexibility. Frequency (but not duration) of formal mindfulness practice was associated with positive wellbeing; however, neither frequency nor duration of formal mindfulness practice was significantly associated with psychological flexibility. Mindfulness teachers will be able to use the present findings to further support their students by reminding them of the benefits as well as normalising some of the challenges of mindfulness practice including falling asleep.

**Electronic supplementary material:**

The online version of this article (10.1007/s12671-018-0951-y) contains supplementary material, which is available to authorized users.

## Introduction

Mindfulness practice has become increasingly popular in recent years, and training in mindfulness skills is now widely available through a variety of courses, workshops, internet programmes and apps, and through one-to-one sessions. Despite this, we know little about how people first begin to practice mindfulness, how often people practice, difficulties they experience, and how people support their practice. There are also uncertainties about how much mindfulness practice is required to produce beneficial effects and regarding the role of informal mindfulness. Understanding these issues is important in order to support the maintenance of mindfulness practice and to promote resilience and wellbeing in the long term.

Mindfulness has been defined as “paying attention in a particular way: on purpose, in the present moment, and non-judgementally” (Kabat-Zinn 2004a, p.4). In other words, choosing to bring the attention to a particular aspect of present experience (such as the breath) without attaching to emotions or thoughts that might arise about or as a result of the experience. This non-judgemental awareness involves acceptance of experience as it is, including those experiences considered to be unpleasant. Non-judgemental awareness is an important aspect of psychological flexibility, which has some overlap with mindfulness. Psychological flexibility is the ability to be fully present and “to change, or persist in, behaviour when doing so serves valued ends” (Biglan et al. 2008, p.142). There is preliminary evidence to suggest that psychological flexibility may be a mechanism of change in mindfulness-based interventions. For example, Duarte and Pinto-Gouveia (2017) found that psychological inflexibility mediated the effects of a mindfulness-based intervention on burnout, compassion fatigue, depression, and stress in a sample of oncology nurses. Processes of mindfulness and acceptance are known to reduce human suffering (Hayes and Plumb 2007), and experiential avoidance is a predictor of poor psychological outcomes (Hayes et al. 1996). The ability to be fully present with experiences, accepting them as they are, can enable people to become aware of their automatic habits and unhelpful reactions, and make more skilful choices. Mindful awareness can be cultivated through both “formal” and “informal” mindfulness practice. While there are no widely agreed definitions of formal and informal practice, formal mindfulness practice can be considered to take place when practitioners specifically set aside time to engage in mindfulness meditation practices such as the body scan, sitting meditation, and mindful movement. Informal mindfulness practice involves weaving mindfulness into existing routines through engaging in mindful moments and bringing mindful awareness to everyday activities, such as mindful eating or mindfully washing the dishes.

Mindfulness-based stress reduction (MBSR; Kabat-Zinn 2004b) and mindfulness-based cognitive therapy (MBCT; Segal et al. 2013) are the most widely available standardised mindfulness-based interventions (MBIs). Both involve eight weekly sessions and recommend daily home practice consisting of both formal and informal mindfulness practice for around 45 min per day. Some studies have reported significant associations between amount of formal home practice and symptom reduction or other outcomes (Crane et al. 2014; Hawley et al. 2014). However, it is not clear how much formal home practice is required to produce benefits. Although daily formal home practice is recommended as part of MBCT, Crane et al. (2014) found that practicing on an average of three or more days per week was sufficient to prevent relapse into depression. Improvements in symptoms have also been reported from evaluations of adapted interventions that involved fewer course sessions, shorter sessions, and shorter formal practices for homework (Cash et al. 2015; Hoge et al. 2013). Furthermore, not all studies examine the relationship between home practice and outcome, some studies report an absence of association, and the precise relationship between home practice and outcome is therefore unclear (Quach et al. 2016; Vettese et al. 2009).

Informal mindfulness involves generalising mindfulness skills to everyday life. For example, Hanley et al. (2015) studied the effect of mindfully washing the dishes and found partial increases in positive affect and partial decreases in negative affect. Hindman et al. (2015) reported greater mindfulness and self-compassion in participants of a mindful stress management-informal (MSM-I) group compared to a waitlist control group. The homework for the MSM-I group involved informal mindfulness practice only. In a study on sleep disturbance, Shapiro et al. (2003) reported participants who practiced more informal mindfulness felt more rested after sleeping (according to self-rated measures of sleep quality). However, other studies do not support a relationship between informal mindfulness practice and outcomes (RCTs such as Crane et al. 2014; Hawley et al. 2014). More recent research has pointed to the key role of informal mindfulness in developing the ability to pay attention to internal and external experiences. Cebolla et al. found frequency of informal practice predicted the “observing” facet of mindfulness as measured by the Five Facet Mindfulness Questionnaire (Cebolla et al. 2017). The evidence regarding the effects of informal mindfulness is therefore unclear, and Crane et al. (2014) suggest that further research is needed before conclusions can be drawn.

Difficulties with formal and informal home practice have been reported by participants of MBIs such as remembering to practice, motivation, finding time, concerns about “getting it right,” and falling asleep during practice (Allen et al. 2009; Hindman et al. 2015; Kabat-Zinn 2004b; Martinez et al. 2015; Moore and Martin 2015; Morgan et al. 2015; Segal et al. 2013). Participants sometimes fall asleep during the longer mindfulness practices such as the body scan, particularly those new to mindfulness meditation (Kabat-Zinn 2004b; Segal et al. 2013). If individuals fall asleep, they are prevented from engaging in the actual practice of mindfulness and this could result in a lack of benefit or even feelings of failure or self-critical thoughts about “not being able to do it” and in some cases, deciding not to continue. It is important to understand more about sleep during practice and particularly attitudes to falling asleep, so that teachers and individuals can discuss the issue more openly, addressing any feelings of failure, and make adjustments where necessary (e.g. practicing at a different time).

Attrition rates for MBIs range from 15 to 30% (Carmody and Baer 2008; Crane and Williams 2010; Kabat-Zinn and Chapman-Waldrop 1988). Increasing understanding of difficulties with mindfulness practice may help to reduce attrition rates as well as providing insights that could inform methods of supporting people to continue to practice mindfulness in the long term. Support for continued formal and informal practice is essential to ensure that health and wellbeing gains achieved from the initiation of mindfulness practice are not lost and to promote further resilience and wellbeing in the long term.

Little is known about how people practicing mindfulness first begin and how they continue to practice, particularly those new to mindfulness or those who did not begin to practice through a standardised course. The present study explores how a large sample of people practicing mindfulness first began to practice and how they continue to practice (both formally and informally), including difficulties and supportive factors. Additionally, the present study investigates the relationship between mindfulness practice and the current wellbeing and psychological flexibility of participants, and whether attending a face-to-face taught course is associated with more frequent formal mindfulness practice. Increased understanding of these areas will provide potential avenues for future research as well as informing the delivery of MBIs and providing insights to support the maintenance of long-term mindfulness practice, thus promoting improvements in health and wellbeing.

## Method

### Participants

Participants were recruited through the social and professional networks of the first author and via online advertising (e.g. mindfulness websites, Facebook, Twitter). Volunteers were requested who currently practice mindfulness or who had practiced mindfulness in the past but were not practicing at the moment and who would be willing to take part in an online questionnaire about their experiences and current wellbeing. No incentives were offered for taking part.

Two hundred and eighteen participants (174 female) took part in the study. The mean age of participants was 44.5 years (SD = 11.7, range 20–77). Fifty-two participants (23.9%) identified themselves as mindfulness teachers with experience of delivering a range of mindfulness-based approaches (formal courses such as MBSR or MBCT, taster sessions or drop-ins, or teaching mindfulness one-to-one). Participant characteristics are shown in Table 1 below.

**Table 1:** Participant characteristics

Level of education	High school = 8 (3.6%)
	College/further education = 26 (11.7%)
	University (undergrad) = 57 (26%)
	University (postgrad) = 127 (57.7%)
Employment status	Working = 164 (75.2%)
	Not working = 11 (5%)
	Retired = 14 (6.4%)
	Student = 21 (9.6%)
	Working and studying = 8 (3.7%)
Experience of mindfulness practice in years	Less than 2 years = 70 (32.1%)
	2–4 years = 78 (35.8%)
	5 years or more = 70 (32.1%)

**Fig. 1:**
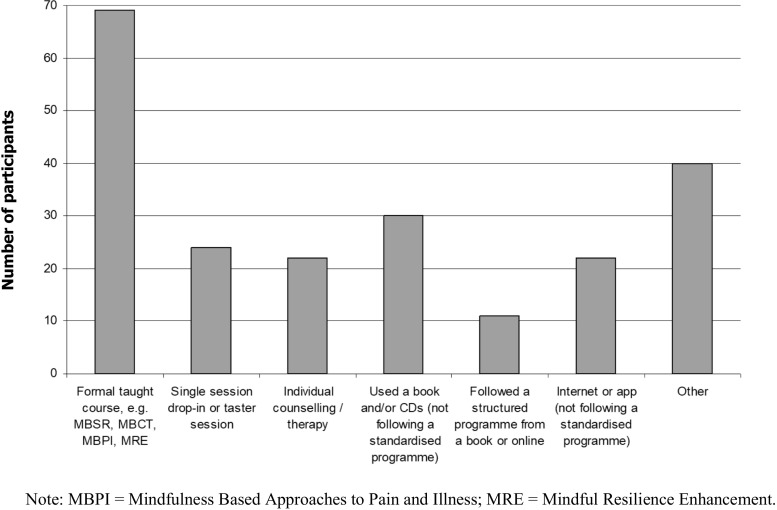
Method of introduction to mindfulness. Note: MBPI = mindfulness-based approaches to pain and illness; MRE = mindful resilience enhancement

Participants were asked to select one of seven options to describe how they first began to practice mindfulness, as illustrated in Fig. 1 below. “Other” was a free-text option and the most common responses in this category were Buddhism (5), other types of meditation (11), through yoga (4), and through work/training (7).

Of the 11 participants who originally followed a structured programme either from a book or online, five went on to attend a face-to-face taught course. Of the 138 participants who did not initially attend a face-to-face taught course or follow a structured programme from a book or online, 24 people later followed a structured course from a book, online, or by telephone, and 41 people later attended a face-to-face taught course. The sample for the present study therefore comprised 115 people (52.8%) who had attended a face-to-face taught course, 30 people (13.8%) who followed a course from a book or online, and 73 people (33.5%) who had not attended or followed any type of structured mindfulness course.

### Procedure

This project included participant involvement in the design of the study. Experienced and novice mindfulness meditators reviewed the study questions and changes were made based on their feedback.

The study was given ethical approval by the University of Derby and participants gave informed consent before participating. Two hundred and seventy-four survey responses were submitted. Data were screened for duplicate IP addresses and any incomplete or duplicate responses were removed, leaving 218 responses eligible for analysis. Several items of categorical data were coded for quantitative analysis including the method of introduction to mindfulness, frequency, and duration of formal and informal mindfulness practice (coded 0 = not practicing to 5 = every day/1 h). Scores for the Acceptance and Action Questionnaire (AAQ-II; Bond et al. 2011) were totalled and raw scores for the Short Warwick Edinburgh Mental Well-Being Scale (SWEMWBS; Stewart-Brown et al. 2009) were transformed to metric scores for analysis.

### Measures

The participant information sheet, consent form, and questionnaires were made available via the online survey software Qualtrics™. The survey was split into sections about different aspects of mindfulness practice beginning with some brief demographic questions followed by a mix of open, closed, and Likert scale questions about how participants first began to practice mindfulness and how they currently practice, including frequency of formal and informal practice, difficulties such as falling asleep during practice and supportive factors for practice. The following options were provided for frequency of formal and informal practice: not practicing, less than once a week, around once a week, once or twice a week, several times a week, and every day; and duration of formal practice: not practicing, 10 min, 20 min, 30 min, 45 min, and 1 h. Participants were not asked to record the duration of informal mindfulness practice as this forms part of everyday activities and it could be difficult to measure the duration accurately. The survey contained separate sections about formal and informal mindfulness practice and the questions relating to current practice can be found in the Supplementary materials (S1). As there are no widely agreed definitions of formal mindfulness practice and informal mindfulness practice, participants were provided with examples of types of practices to help guide their responses. For example, body scan, sitting practice, and being mindful while washing the dishes, while driving, or eating. Informal practice was also referred to as “every day mindful moments.” Free-text “comments” fields were included in the survey as often as possible so that participants could expand on and clarify their responses, particularly if the phrasing of a question did not fit with their experience of mindfulness.

Two standardised scales were also included. The Short Warwick Edinburgh Mental Well-Being Scale is a seven-item scale consisting of positively phrased items that are scored 1–5 and summed to give an index of mental wellbeing (Stewart-Brown et al. 2009). Higher scores indicate greater mental wellbeing (score range 7–35). SWEMWBS meets the Rasch Measurement model for construct validity (Stewart-Brown et al. 2009). In the current study, Cronbach’s *α* was 0.86 for SWEMWBS. The Acceptance and Action Questionnaire (AAQ-II) measures aspects of psychological flexibility, particularly acceptance and experiential avoidance (Bond et al. 2011). The AAQ-II is a psychometrically evaluated scale with seven items that are scored 1–7. Lower scores indicate greater acceptance and action, and higher scores indicate greater experiential avoidance (score range 7–49). Although it was not designed as a diagnostic tool, comparisons with other scales suggest that AAQ-II scores of 24–28 and higher indicate psychological distress (Bond et al. 2011). In the current study, Cronbach’s *α* was 0.90 for the AAQ-II.

### Data Analyses

Analyses were conducted using SPSS v22. Data were not normally distributed, so Mann-Whitney was used to test for differences in frequency of formal mindfulness practice between those who had attended a face-to-face taught mindfulness course and those who had not. Hierarchical regressions tested for relationships between mindfulness practice and wellbeing. SWEMWBS and AAQ-II scores were entered as dependent variables; frequency of formal mindfulness practice, duration of formal mindfulness practice, and frequency of informal mindfulness practice were entered on the first step and two potentially confounding variables were entered on the second step. These variables were mindfulness teacher status (yes or no) and duration in years of mindfulness practice. It was suspected that these variables may affect wellbeing and psychological flexibility. Several scores were missing from the AAQ-II and SWEMWBS responses and so ten participants were excluded from analyses involving these measures. Three participants had one score missing, which was resolved using imputation through interpolation and extrapolation.

Responses to open-ended questions regarding sleep during practice and support for practice were analysed using inductive content analysis (Elo and Kynga 2008). Data were coded and categorised by the first author and the second author corroborated the coding and categories. The frequency of coded categories was then recorded.

## Results

### Beginning to Practice Mindfulness

The 145 participants who had attended a face-to-face course or followed a course from a book or online provided further information about the course and their experience. Sixteen participants (11%) attended courses delivered by the NHS: 14 face-to-face and two online. Sixty-eight people (46.9%) paid for their face-to-face taught course and eight people (5.5%) paid for their online course. Most participants (90; 62.1%) attended or followed courses consisting of eight sessions. One hundred and thirty-five participants answered the question “Why did you attend the mindfulness course?” Responses were categorised and can be found in Table 2 below (some participants gave more than one reason).

**Table 2:** Reasons for attending the mindfulness course

	Attended a face-to-face taught course (114 respondents)	Followed a course from a book or online (21 respondents)
Curiosity/personal interest	30	5
Personal development	16	6
Health reasons	46	7
Work/CPD	24	2
To train to teach	13	1

Falling asleep during practice was common: 62 people (42.8%) reported falling asleep during practices in the course sessions and 49 of these were attendees of a face-to-face course. Of these 62 participants, 54 (87.1%) said they fell asleep during the body scan (42 were attendees of a face-to-face course). Ninety-six people (66.2%) reported falling asleep during their home practice (77 were attendees of a face-to-face course). One hundred and thirty-nine participants answered the question about home practice and 22 (15.8%) said they completed the home practice all of the time, 72 (51.8%) said they completed the home practice most of the time, and 34 (24.5%) said they completed the home practice some of the time. Eighteen participants (12.9%) said they did not feel able to discuss difficulties with home practice with the course teachers compared to 107 (77%) who did feel able to discuss this. Fifty-nine participants (53 who attended a face-to-face taught course) were offered a reunion/top-up session, and 35 (32 who attended a face-to-face taught course) attended a reunion/top-up session. One hundred and thirty-five people (96.4%) said they found the mindfulness course helpful, compared to five (3.6%) who did not (five people did not answer this question). When asked if they thought the mindfulness course made any difference to their life, 141 participants answered as follows: significant positive change: 90 (63.8%), some positive change: 46 (32.6%), no change: three (2.1%), and some negative change: two (1.4%).

### Current Practice

**Fig. 2:**
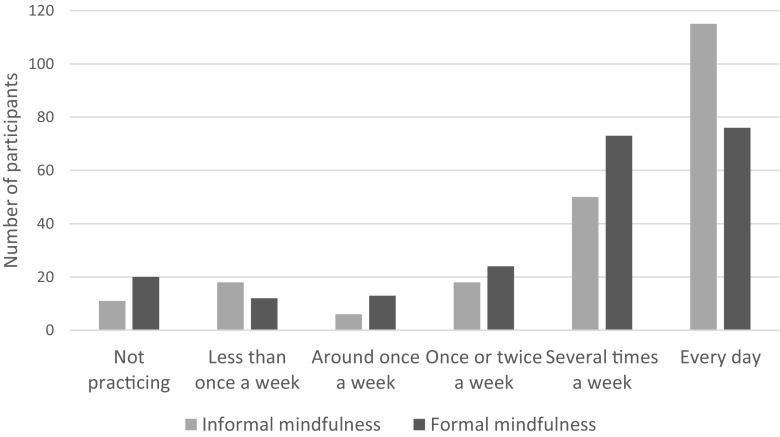
Frequency of formal and informal mindfulness practice

Of the 218 participants, 198 reported that they were currently practicing formal mindfulness, and 207 reported that they were currently practicing informal mindfulness. In a free-text comment field, some participants gave examples of their informal mindfulness practice. These included washing the dishes, eating, driving, brushing teeth, walking the dog, drinking coffee, and watching a wild bird or flower. Self-reported frequency of formal and informal mindfulness practice is illustrated in Fig. 2 below. As can be seen, most participants reported practicing several times a week or every day, indicating a regular practice. Twenty-four participants (12.1%) practiced formal mindfulness for 45 min or longer at a time, 56 participants (28.3%) practiced for 30 min, 68 participants (34.3%) practiced for 20 min, and 50 (25.3%) practiced for 10 min at a time.

For the purpose of inferential statistics, frequency of practice was coded from 0 (not practicing) to 5 (practicing every day) and a Mann-Whitney test revealed that there was no significant difference in the frequency of formal mindfulness practice between those who had attended a face-to-face taught course (Mdn = 4) and those who had not attended a face-to-face taught course (Mdn = 4), *U* = 5346.50, *z* = − 1.29, ns, *r* = − 0.09.

### Mindfulness Practice, Wellbeing, and Psychological Flexibility

The mean wellbeing (SWEMWBS) score for the sample of 208 participants was 23.75 (SD = 3.46, range 15.32–35), and the mean psychological flexibility (AAQ-II) score was 19.08 (SD = 7.60, range 7–42). These scores are consistent with norms of 23.7 for SWEMWBS (Ng Fat et al. 2016) and 18.51 for the AAQ-II (Bond et al. 2011). All three aspects of mindfulness practice significantly positively correlated with each other, and wellbeing was significantly negatively correlated with psychological flexibility (see Table 3). Negative values are due to lower AAQ-II scores indicating greater acceptance and higher scores indicating greater experiential avoidance.

**Table 3:** Correlations among study variables

	1	2	3	4	5
1. SWEMWBS	–	–	–	–	–
2. AAQ-II	− .607**	–	–	–	–
3. Frequency of formal mindfulness practice	.298**	− .161*	–	–	–
4. Duration of formal mindfulness practice	.242**	− .162*	.467**	–	–
5. Frequency of informal mindfulness practice	.330**	− .272**	.333**	.196*	–

*p < .05

**p < .001

Hierarchical regression tested the relationship between mindfulness practice and wellbeing. For model 1, frequency of formal mindfulness practice (*t*(204) = 2.098, *β* = .159, SE = .165, *B* = .345, *p* = .037) and frequency of informal practice (*t*(204) = 3.72, *β* = .254, SE = .154, *B* = .574, *p* < .001) were significantly related to wellbeing, and frequency of informal mindfulness was the most important variable in this model. Duration of formal mindfulness practice (*t*(204) = 1.62, *β* = .118, SE = .212, *B* = .344, *p* = .106) was not significantly related to wellbeing. Model 2 included mindfulness teacher status and duration of mindfulness practice in years. Neither of these variables were significantly related to wellbeing (teacher status: *t*(202) = 1.49, *β* = .110, SE = .598, *B* = .892, *p* = .138; years of practice: *t*(202) = .459, *β* = .033, SE = .037, *B* = .017, *p* = .647). However, including these variables affected the relationships between wellbeing and frequency of formal mindfulness practice (*t*(202) = 1.96, *β* = .149, SE = .166, *B* = .324, *p* = .052) and wellbeing and duration of formal mindfulness practice (*t*(202) = 1.25, *β* = .093, SE = .217, *B* = .272, *p* = .212), resulting in there no longer being a significant relationship between wellbeing and frequency of formal mindfulness practice. In model 2, frequency of informal mindfulness practice was the only variable significantly related to wellbeing (*t*(202) = 3.18, *β* = .222, SE = .158, *B* = .503, *p* = .002). The combined influence of mindfulness practice (model 1) accounted for 15.9% of the variation in wellbeing (*R*^2^ = .159, adjusted *R*^2^ = .147). This increased slightly to 17.3% (*R*^2^ = .173, adjusted *R*^2^ = .153) when the other variables were added (model 2). Although ANOVA results indicated that both models were significant predictors of wellbeing (*p* = .000), model 1 (*F* = 12.890) was a better predictor than model 2 (*F* = 8.477). Both models showed small effect sizes (model 1: .198; model 2: .209).

Hierarchical regression also tested the relationship between mindfulness practice and psychological flexibility. For model 1, frequency of informal mindfulness practice (*t*(204) = − 3.395, *β* = − .241, SE = .352, *B* = − 1.197, *p* = .001) was significantly related to psychological flexibility. Frequency of formal practice (*t*(204) = − .446, *β* = − .035, SE = .376, *B* = − .168, *p* = .656) and duration of formal mindfulness practice (*t*(204) = − 1.296, *β* = − .098, SE = .484, *B* = − .627, *p* = .197) were not significantly related to psychological flexibility. Again for model 2, neither mindfulness teacher status (*t*(202) = − 1.161, *β* = − .089, SE = 1.36, *B* = − 1.580, *p* = .247) nor years of mindfulness practice (*t*(202) = − 1.478, *β* = − .111, SE = .084, *B* = − .124, *p* = .141) were significantly related to psychological flexibility. Adding these variables to the model did not significantly affect the sizes of the relationships between psychological flexibility and the three aspects of mindfulness practice. Again, frequency of informal mindfulness practice was the only practice variable significantly related to psychological flexibility (*t*(202) = − 2.763, *β* = − .201, SE = .360, *B* = − .995, *p* = .006). The combined influence of mindfulness practice (model 1) accounted for 8.7% of the variation in psychological flexibility (*R*^2^ = .087, adjusted *R*^2^ = .074). This increased to 11.2% (*R*^2^ = .112, adjusted *R*^2^ = .090) when the other variables were added. Although ANOVA results indicated that both models were significant predictors of psychological flexibility, model 1 (*F* = 6.494, *p* < .001) was a better predictor than model 2 (*F* = 5.094, *p* < .001). However, the models only explained that a small amount of the variance and effect sizes were small (model 1: .095; model 2: .126).

### Challenges and Support for Mindfulness Practice

The 198 participants practicing formal mindfulness were asked which practices from those listed they completed regularly. The number of participants that selected each practice is as follows: body scan (89, 44.9%), sitting practice (125, 63.1%), breathing space (112, 56.6%), mindful movement (52, 26.3%), all of the above (31, 15.7%), and other (34, 17.2%). In the free-text option for “Other,” participants stated a variety of additional practices including mindfulness of sound and loving-kindness meditation. Seventy-six participants (38.4%) said that there were some practices they disliked or found difficult and so did not do. The most common of those listed by participants were the body scan (27, 35.5%) and mindful movement or walking (18, 23.7%). Participants were then asked to select the options that best described their experiences of mindfulness practice, and responses were as follows: easy (57, 28.8%), interesting (97, 49%), practice reluctantly (40, 20.2%), irritating (31, 15.7%), practice willingly (123, 62.1%), difficult (72, 36.4%), enjoyable (100, 50.5%), boring (27, 13.6%), relaxing (127, 64.1%), ok (45, 22.7%), it is what it is (90, 45.5%), and blissful (33, 16.7%). There were a number of free-text comments about practice varying from day to day. Other comments touched on the usefulness of practice as well as difficulties with practice including perceptions of how practice “should be,” such as “can occasionally be hard to stay sitting if the mind is pre-occupied - useful still to sit and be with boredom or irritation and come back to focusing anchor.” “Can sometimes make me aware of things I hadn’t known I was feeling, which can annoy me when I don’t feel ready to deal with them emotionally.” “Sometimes guilt-inducing because I think I’m not doing them right and I should be better by now.” “Each meditation is different. Sometimes being with the uncomfortable is challenging. Whatever the moment brings … can be blissful and easy one day then difficult and uncomfortable the next.”

### Falling Asleep During Mindfulness Practice

Of the 198 participants currently practicing formal mindfulness, 112 (56.6%) reported falling asleep during practice. Seventy of these (62.5%) said that they did not fall asleep regularly and 11 (9.8%) said that they had a medical condition that they thought made them more likely to fall asleep during practice. Participants reported falling asleep most frequently during the body scan (67, 57.8%) and breathing/sitting practice (23, 19.8%). One-hundred and five participants answered the open question “How do you feel about falling asleep?” Responses were analysed in line with Elo and Kynga (2008) and categorised as follows: intended to/find it helpful for getting to sleep (6, 5.7%), positive response (6, 5.7%), accepting response (71, 67.6%), negative response (15, 14.3%), combination of acceptance, and negative response (7, 6.7%). Some participants whose responses fit within the “positive” or “accepting” categories commented on the beneficial effects of falling asleep, e.g. “Fine, it has helped me to learn to fall asleep during bouts of insomnia!” and “Love it. I always wake rested even more than if I lay down for a rest.” When responses to questions about sleep were broken down according to how people first began to practice mindfulness, the types of response given appeared to be proportionate across the groups. A breakdown can be found in Supplementary material (S2).

### Factors that Support Participants to Practice Mindfulness

Participants were asked to select all that applied from a range of options to describe how their current practice is supported. One-hundred and ninety-six participants responded as follows: CD (72, 36.7%), app (69, 35.2%), self-guided (143, 73%), guided by others (21, 10.7%), practice in a group with guidance (35, 17.9%), and practice in a group without guidance (13, 6.6%). Responses to the open question of what is or would be supportive were analysed in line with Elo and Kynga (2008); four main categories of response were identified. Some responses fit within more than one category. The categories were “practical resources” (97), “time/routine” (36), “support from others” (85), and “attitudes and beliefs” (29).

#### Practical Resources

Participants stated tools such as apps, CDs, and emails from websites helped to support practice. Evidence and reminders that mindfulness can be helpful were seen as supportive. Some participants created their own practice reminders in the home or using cues from their environment. For example: “a reminder bracelet, pic on my desk, the school bell is when I breathe etc.” “Following mindful accounts on twitter & newsletters.” “An understanding of the basis for mindfulness and examples of success in using mindfulness more than it made me feel better.” “CDs, downloads, and apps are great, a nice voice is important either male or female, good books with website/links are very useful.”

#### Time/Routine

Participants highlighted the importance of finding time to practice and stated that this wasn’t always easy. Being able to incorporate practice into a daily routine or form a routine for practice was also important.

#### Support from Others

Being part of a mindfulness community was highly valued. Fourteen participants explicitly stated that they attend regular group practice sessions or course refresher/reunion sessions in order to support their practice. Contact with mindfulness teachers, attending workshops, group practice sessions, and talking to others who practice all helped to support ongoing mindfulness practice. Support from friends, family, and the workplace was also seen as important. For example: “I buddy up with a friend and we text each other when we have completed a formal practice. I taught her mindfulness and now offer informal supervision. This monthly connection keeps me on track ….” “Regular opportunities to formally practice with others and share ideas or experiences about practice. It inspires and reinvigorates.” “Mindfulness at workplace, in a work culture where mindfulness is valued.” “More connection with community of other practitioners.”

#### Attitudes and Beliefs

Some participants stated that their own beliefs and experience supported them to continue to practice; feelings during and following practice and feeling a benefit. Attitudes of acceptance and kindness were also important, particularly if practice had lapsed. For example: “the experience of life being easier, more fulfilling, having resources to cope with difficulty when I practice.” “Knowing the benefits I gain from doing. Knowing that if I stop it’s like a muscle and weakens. Knowing that I can always begin again, which is very kindly and forgiving.” “Knowing that you can do anything mindfully and you can’t get it wrong.” “The encouragement to trust that every moment can be mindful without having to use a formal approach.”

## Discussion

The current study explored how participants first began to practice mindfulness and how they continued, difficulties they experienced, and how they supported their ongoing practice. The study also investigated associations between formal and informal practice and wellbeing and psychological flexibility.

### Beginning to Practice Mindfulness

The participants in this study first began to practice mindfulness in a wide variety of ways, which reflects the range in methods of delivery of mindfulness outside health services. Remarkably, almost half the sample had not attended a face-to-face taught course.

In line with previous research (Hindman et al. 2015; Martinez et al. 2015; Moore and Martin 2015; Morgan et al. 2015), many participants in the present study who took part in a mindfulness course experienced difficulties with home practice such as staying awake or being unable to complete all of the recommended home practice. Although only a small number of people in this study said they did not feel able to discuss home practice difficulties with the course teacher, this is a concern. According to Allen et al. (2009), MBCT therapists are likely to be more effective if they have an awareness of what creates struggle, and this is equally true for professionals delivering other types of MBIs besides MBCT. The ability to model compassion and acceptance as well as drawing on the teachers own experience of practice could help to encourage those learning mindfulness skills to talk about any difficulties they experience and ensure these are not perceived as “failures.”

### Mindfulness Practice, Wellbeing, and Psychological Flexibility

Most of the participants in this study were continuing to practice mindfulness, and more participants were practicing informal mindfulness on a daily basis than formal mindfulness. The results of the multiple regression suggest that frequency of informal mindfulness is more important for wellbeing and psychological flexibility than frequency or duration of formal mindfulness practice. Being a mindfulness teacher and the number of years of mindfulness practice mediated the relationship between frequency of formal mindfulness practice and wellbeing; however, frequency of informal mindfulness remained significantly related. Furthermore, status as a mindfulness teacher was not significantly related to wellbeing or psychological flexibility, suggesting little difference between the teachers and non-teachers who participated in this study. This could suggest that there is simply little difference in the levels of wellbeing and psychological flexibility between the teachers and non-teachers, or there could be complex confounding factors. For example, baseline levels of wellbeing and psychological flexibility of participants from when they first began practicing mindfulness or teaching mindfulness are not known and this could be a factor in the current wellbeing of participants. Additionally, those with higher levels of trait or dispositional mindfulness may be more aware of a lack of wellbeing and those with lower levels of mindfulness may not notice a lack of wellbeing. Dispositional mindfulness is discussed in more detail below.

The significance of informal mindfulness found in this study may be explained in part by the recent findings from Cebolla et al. (2017), where frequency of informal mindfulness practice predicted the “observing” facet of mindfulness. This ability to pay attention to experience is a key mindfulness skill and as mentioned earlier, overlaps with psychological flexibility. However, the statistical models in this study only explained a small amount of the variance and effect sizes were small, so other factors may have influenced the results. Nevertheless, the findings point to the importance of informal mindfulness and are consistent with previous research about the beneficial effects of informal mindfulness practice (Hanley et al. 2015; Hindman et al. 2015). Further research regarding the role of informal mindfulness is encouraged.

Levels of dispositional mindfulness and the individual differences of participants could have affected the findings. Dispositional mindfulness has been defined as “the tendency to express mindful attitudes and behaviours in everyday life” (Hanley et al. 2017). Dispositional mindfulness can increase as a result of mindfulness practice and can in turn improve wellbeing (Carmody and Baer 2008). However, there can be individual differences in levels of dispositional mindfulness, and individuals who do not practice mindfulness may be mindful in their everyday life (Hanley et al. 2017). Additionally, individual differences in personality may be a factor in who begins and continues to practice mindfulness, as well as in the resultant effects of practice. Dispositional mindfulness and personality factors were not measured as part of the current study and the inclusion of a measure such as the Five Facet Mindfulness Questionnaire (Baer et al. 2006) or the Big Five Inventory (John et al. 1991) could have been beneficial in further understanding the findings.

Furthermore, the relationship between mindfulness practice and outcome is complex, and Crane et al. (2014) highlighted a lack of evidence regarding the confounding factors affecting home practice and outcome, suggesting that perceptions of treatment plausibility could be a factor. This was reflected in the qualitative data from the current study regarding support for practice. Participants reported that knowledge of the benefits of mindfulness practice (either from books or research evidence, or from their own experience of feeling positive effects) supported them to continue practicing. Moore and Martin (2015) also found beliefs about the benefits of practice to be a motivator to practice. The perception of benefit as well as other factors identified from the qualitative data (discussed below) in this study could therefore not only have supported people to practice but also could potentially have contributed to the wellbeing of participants.

### Challenges and Support for Mindfulness Practice

Participants’ descriptions of the experience of mindfulness practice were wide-ranging, from difficult to blissful, and many participants said that their practice varied from day to day. In line with previous research (Allen et al. 2009), some participants commented on “getting it right” which implied a sense of there being something to achieve as well as wanting things to be other than they are. These findings highlight one of the difficulties regarding the intention of mindfulness practice in that there is no ultimate goal or state to achieve other than to be aware of things as they are, and the experience of practice is ever changing. This again points to the skills required by a mindfulness teacher, as well as the teacher’s own experience of mindfulness practice, to support participants to manage such challenges.

More than half of the participants who were practicing formal mindfulness had experienced falling asleep during practice, which suggests that this is not unusual or uncommon. Although content analysis revealed most participants responded to this experience with acceptance, some participants had a negative response. Knowing that falling asleep is a common occurrence should help to open up discussions about adjustments that can be made to support people to stay awake during practice, while also cultivating an attitude of acceptance that sometimes the body simply needs to sleep. Indeed, six of the 112 participants who reported falling asleep during practice said that it was their intention to fall asleep, and other participants commented on the beneficial effects of falling asleep such as helping with insomnia and feeling more rested (more so than if they take a nap). This suggests that increased sleep was sometimes viewed as a positive outcome. The acceptance of sleep by participants in this study could be indicative of a general attitude of acceptance which could be a contributory factor to the AAQ-II scores, discussed further below.

Practical resources, time/routine, support from others, and attitudes and beliefs were identified as common themes that supported home practice. Technology (e.g. apps, email reminders) and cues in the environment helped participants to remember to practice as well as form a routine. These findings are in line with suggestions for practice support made by Hindman et al. (2015) in response to the home practice difficulties experienced by participants in their study.

Support from friends, family, and others practicing mindfulness was an important factor in continuing to practice, and some participants attended regular group practice sessions or course refresher/reunion sessions. This support and being part of a “mindfulness community” could also have been a confounding factor that contributed to wellbeing. For example, one participant stated: “I really enjoyed the class mostly because of the interactions with other people. I’d like to be a part of a regular formal practice community.” For this participant, the social aspect of attending a course appears to be more important than the practice itself. Continuing to be part of a community, in contact with others who practice, could therefore impact on wellbeing.

Knowledge and beliefs about practice were also identified as supportive factors. Embedded in these qualitative responses were attitudes of trust, beginner’s mind, and acceptance. These attitudes were both implied and explicit in the language, for example: “Knowing that I can always begin again, which is very kindly and forgiving” and “The encouragement to trust that every moment can be mindful.” Kabat-Zinn (2004b) described seven attitudes that underpin mindfulness practice: non-judging, patience, a beginner’s mind, trust, non-striving, acceptance, and letting go. These attitudes are interconnected and form the foundation of mindfulness practice, helping to determine its long-term value to individuals (Kabat-Zinn 2004b). So, as well as just “doing” mindfulness practice, these attitudinal factors are important. This could provide a partial explanation for the AAQ-II results. The AAQ-II measures psychological flexibility with a particular emphasis on experiential avoidance and acceptance. Thus, while mindfulness practice in itself is important, the cultivation of these attitudinal foundations of mindfulness could have had particular bearing on AAQ-II scores.

### Limitations and Future Research

The majority of participants in this study were female; however, a larger proportion of female participants is not uncommon in mindfulness research conducted online (for example Wahbeh et al. 2014). Participants were also highly educated; however, the level of education of participants is consistent with a recent study from Cebolla et al. (2017), which had a sample of 365 participants, 89.8% of whom were university graduates. Methods of increasing access to MBIs and mindfulness research to more diverse populations are therefore to be encouraged. Additionally, it was not possible to ascertain the proportion of participants recruited to the study via social media, and there could be differences among participants according to the method of recruitment.

The study is limited by the lack of exploration of the processes of focused attention and open monitoring during mindfulness practice. Due to the complexities of mindfulness practice, the study questionnaire could also have benefited from the inclusion of a measure of dispositional mindfulness such as the Five Facet Mindfulness Questionnaire (Baer et al. 2006) or a measure of personality factors such as the Big Five Inventory (John et al. 1991).

Although sleep during practice was explored as part of the present study, the questionnaire did not include items about sleep patterns of the participants. Including items about sleep patterns of participants could have helped with the exploration of attitudes to falling asleep during practice. Additionally, the study is limited by the collection of data at a single time point and it would have been valuable to look at experiences of falling asleep and factors such as wellbeing, psychological flexibility, and dispositional mindfulness over time and whether these change in relation to changes in patterns of mindfulness practice. This would be particularly valuable in relation to the role of informal mindfulness practice, which is yet to be fully understood and requires further study.

Experiences of mindfulness were wide-ranging, and it was not possible to capture the intricacies of such experiences in this survey. There may be inaccuracies with the amount of practice that was reported, particularly informal mindfulness, which other researchers have stated is difficult to capture (Crane et al. 2014). Informal mindfulness practice, while undoubtedly important, is also complex. Participants may have had different interpretations of informal mindfulness practice, and this study did not explore the relationship between formal and informal practice and how individuals may begin to weave mindfulness into their lives more effortlessly as a result of regular formal practice. Future research should consider the effects of informal mindfulness on study outcomes, particularly across a number of timepoints, as well as exploring the relationship between formal and informal mindfulness practice.

## Electronic Supplementary Material

ESM 1 (PDF 201 kb)

ESM 2 (PDF 12 kb)
