# Supplementary material for: An Exploration of Formal and Informal Mindfulness Practice and Associations with Wellbeing
Source: Mindfulness (N Y). 2018 May 21;10(1):89–99. doi: 10.1007/s12671-018-0951-y (PMC6320743; doi:10.1007/s12671-018-0951-y)
Supplement: Supplementary file 1 — (PDF 201 kb) [file 12671_2018_951_MOESM1_ESM.pdf]

How do people practice mindfulness: survey questions

Current formal mindfulness practice:

Do you currently practice formal mindfulness? (e.g. body scan, sitting practice, breathing space, mindful movement) Yes / No
Comments: ____

If 'No' is selected:

1. Why are you no longer practicing? Select all that apply:

Lack of time	Hadn't formed a habit	Got out of the habit
I didn't find it helpful	I decided it wasn't for me	Other stressors
I felt worse during or after practice	Loss of teacher support	
Loss of support of the group setting		

Other: ____________________________

2. Is there anything you can think of that would have supported you to continue with formal mindfulness practice? ________________________________

If 'Yes' is selected:

1. How often do you practice? Every day / several times a week / once or twice a week / around once a week / less than once a week

2. How long **on average** does your practice session last? 1 hour / 45 minutes / 30 minutes / 10 minutes
Comments: _____________________________

3. How is your practice supported (select all that apply): CD / app / self-guided / guided by others / practice in a group with guidance / practice in a group without guidance
Comments: _______________________________________

4. If you aren't practicing as regularly as you used to, or as you would like, is there anything you can think of that would support you to practice more regularly?
____

5. Which practices do you do most regularly?

body scan	sitting practice	breathing space	mindful movement
all of the above	other		

Comments: ____

6. How would you describe your experience of these practices? We understand that your experience of practice may change from day to day so please select **all** that apply:

Easy	difficult	enjoyable	boring	practice reluctantly	interesting
irritating	relaxing	it is what it is	blissful	practice willingly	ok

Comments: ____________________________

7. Are there any practices you dislike or find difficult, and so do not do? Yes / No

8. If yes, which practices? ____________________________

Informal mindfulness:

1. Do you engage in everyday mindful moments? For example being mindful while washing the dishes, while driving, or eating. Yes / No

2. If yes, how often? Every day / several times a week / once or twice a week / around once a week
Comments: ____
