# Supplementary material for: An Exploration of Formal and Informal Mindfulness Practice and Associations with Wellbeing
Source: Mindfulness (N Y). 2018 May 21;10(1):89–99. doi: 10.1007/s12671-018-0951-y (PMC6320743; doi:10.1007/s12671-018-0951-y)
Supplement: Supplementary file 2 — (PDF 12 kb) [file 12671_2018_951_MOESM2_ESM.pdf]

How do people practice mindfulness: sleep data, current practice

Group 1: attended a face-to-face taught course (N=115)
Group 2: followed a course from a book or online (N=30)
Group 3: did not attend or follow a structured course (N=73)

Of the 198 participants currently practicing formal mindfulness 112 (56.6%) reported falling asleep during practice:
Group 1: 63
Group 2: 20
Group 3: 29

Do you fall asleep regularly? No:
Group 1: 39
Group 2: 10
Group 3: 21

Which practices do you fall asleep during?
Body scan:
Group 1: 43
Group 2: 12
Group 3: 12

Breathing/sitting practice:
Group 1: 10
Group 2: 3
Group 3: 10

How do you feel about falling asleep?

	Grp 1	Grp 2	Grp 3
Intended to/find it helpful for getting to sleep	2	1	3
Positive response	1	1	3
Accepting response	39	12	19
Negative response	13	1	3
Combination of acceptance and negative response	5	1	1
